# Abatacept to induce remission of peanut allergy during oral immunotherapy (ATARI): protocol for a phase 2a randomized controlled trial

**DOI:** 10.3389/fmed.2023.1198173

**Published:** 2023-06-28

**Authors:** Camille Braun, Pauline Azzano, Florence Gingras-Lessard, Émilie Roy, Kathryn Samaan, François Graham, Louis Paradis, Anne Des Roches, Philippe Bégin

**Affiliations:** ^1^Department of Pediatrics, Section of Allergy and Clinical Immunology, Centre Hospitalier Universitaire Sainte-Justine, Montreal, QC, Canada; ^2^Department of Pediatrics, Pneumology, Allergy, Cystic Fibrosis, Hôpital Femme Mère Enfant, Hospices Civils de Lyon, Bron, France; ^3^Centre International de Recherche en Infectiologie, INSERM U1111, CNRS UMR 5308, Université Lyon 1, ENS de Lyon, Lyon, France; ^4^Department of Pediatric Hepatogastroenterology and Nutrition, Hôpital Femme Mère Enfant, Hospices Civils de Lyon, Bron, France; ^5^Department of Medicine, Section of Allergy and Clinical Immunology, Centre Hospitalier de l'Université de Montréal, Montreal, QC, Canada

**Keywords:** food allergy, oral immunotherapy (OIT), sustained unresponsiveness, abatacept, CTLA-4, peanut allergy, desensitization

## Abstract

**Context:**

While oral immunotherapy (OIT) has been shown to promote the remission of mild peanut allergy in young children, there is still an unmet need for a disease-modifying intervention for older patients and those with severe diseases. In mice models, abatacept, a cytotoxic T lymphocyte-associated antigen-4 (CTLA-4) immunoglobulin fusion protein, has been shown to promote immune tolerance to food when used as an adjuvant to allergen immunotherapy. The goal of this study is to explore the potential efficacy of abatacept in promoting immune tolerance to food allergens during OIT in humans.

**Methods:**

In this phase 2a proof-of-concept study (NCT04872218), 14 peanut-allergic participants aged from 14 to 55 years will be randomized at a 1:1 ratio to abatacept vs. placebo for the first 24 weeks of a peanut OIT treatment (target maintenance dose of 300 mg peanut protein). The primary outcome will be the suppression of the OIT-induced surge in peanut-specific IgE/total IgE at 24 weeks, relative to the baseline. Sustained unresponsiveness will be assessed as a secondary outcome starting at 36 weeks by observing incremental periods of peanut avoidance followed by oral food challenges.

**Discussion:**

This is the first study assessing the use of abatacept as an adjuvant to allergen immunotherapy in humans. As observed in preclinical studies, the ability of abatacept to modulate the peanut-specific immune response during OIT will serve as a proxy outcome for the development of clinical tolerance, given the small sample size. The study will also test a new patient-oriented approach to sustained tolerance testing in randomized controlled trials.

## 1. Introduction

### 1.1. Context of the study

Oral immunotherapy (OIT) is a new treatment for food allergy (FA) (1). It consists of the regular ingestions of the allergen in a medically supervised manner to achieve a state of desensitization, defined as an increase in the patient's reactivity threshold while on therapy. Desensitization can be achieved in most patients with success rates ranging from 60 to 100% (2–6). It offers protection from accidental ingestion and improvement in quality of life (7). Over time, OIT can also lead to clinical remission, which is defined as a lack of clinical reaction to a food allergen after active therapy has been discontinued, thus indicating a modification of the underlying disease (8). It is more likely to be achieved in younger children with low levels of food-specific immunoglobulin E (IgE) and is seldom observed after the age of 9 years (9, 10). In other words, OIT appears to have a limited ability to induce oral tolerance once immune memory has been firmly established. Thus, there is an unmet need for an intervention that can target the patient's immune memory to the allergen.

### 1.2. Memory reactivation during OIT

FA memory is mediated by B and T memory cells in lymphoid organs as well as by long-lived IgE-producing plasma cells that can persist for years in the bone marrow (11–13).

During the early phase of OIT, exposure to the allergen results in the reactivation of memory B cells that are dependent on type-2 memory T cells (Th2 cells) (14), which leads to the generation of new IgE-producing plasma cells and the expansion of the allergen-specific B-cell repertoire through somatic hypermutation (15). This phenomenon is reflected in the 1st month of therapy by a sudden rise in allergen-specific IgE (sIgE) (16). The intensity of the sIgE surge has been shown to inversely correlate with the likelihood of developing clinical remission, further suggesting its value as a proxy measure for immune memory reactivation (9, 17). Clinical remission is also typically associated with allergen-specific T-cell transition from a type 2 phenotype to one of immune regulation or exhaustion (18).

### 1.3. Effect of CTLA-4 and abatacept on T cells and plasma cells

Abatacept is a cytotoxic T lymphocyte-associated antigen (CTLA-4)-immunoglobulin fusion protein approved by the U.S. Food and Drug Administration (FDA) and Health Canada for adults with psoriasis and rheumatoid arthritis as well as for children of 6 years and older with juvenile idiopathic arthritis. Abatacept interferes with the delivery of the costimulatory signal required for T-cell activation and plasma cell survival (19).

**Figure 1:**
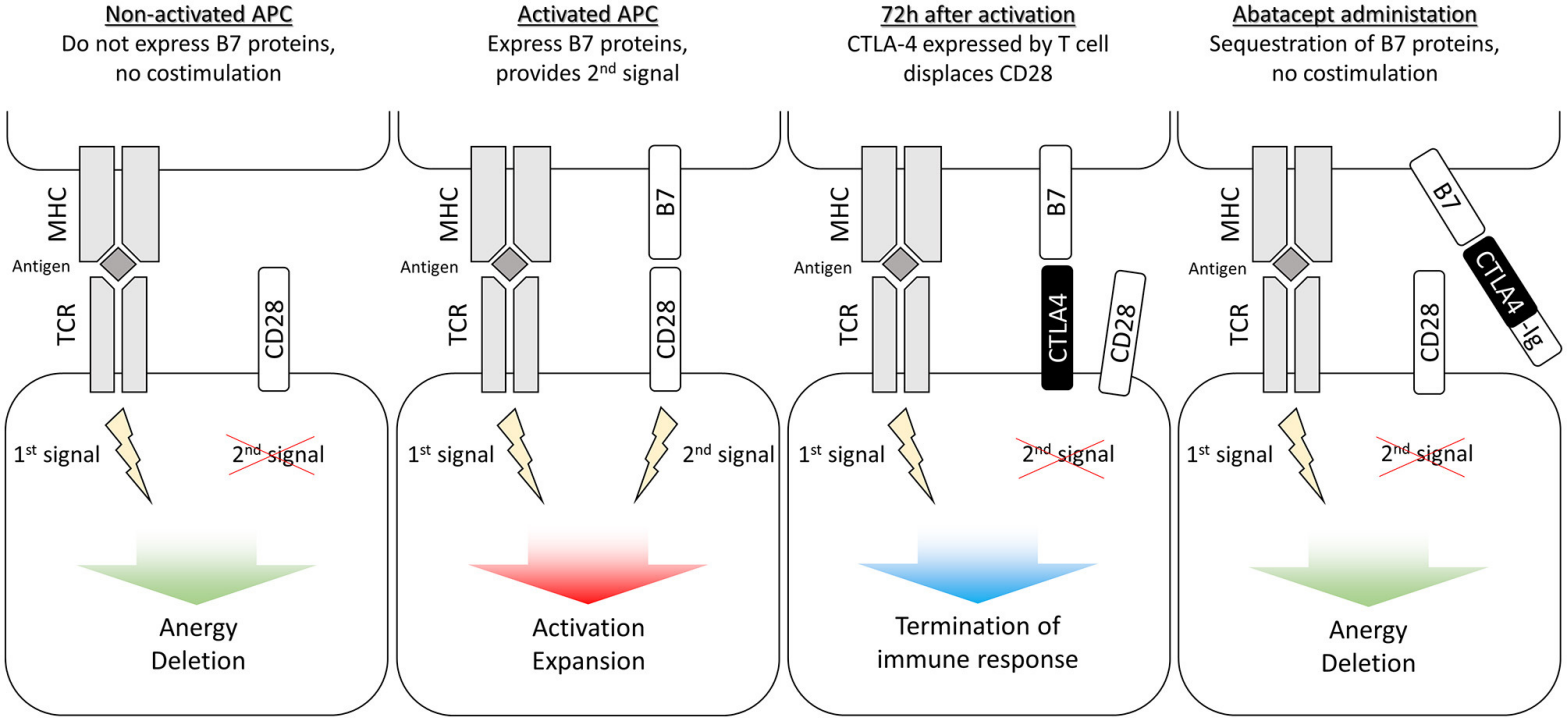
Second signal blockade in T cells. APC, antigen-presenting cell; CTLA-4, cytotoxic T lymphocyte-associated antigen-4; MHC, major histocompatibility complex; TCR, T-cell receptor.

T cells require two distinct signals from antigen-presenting cells (APCs) in order to activate (20). The first signal is delivered to the T-cell receptor (TCR) by a major histocompatibility complex (MHC) carrying the antigen and is responsible for immune specificity. The second signal is mediated by co-stimulatory receptors, which validate that an immune response should indeed be mounted against that antigen. It is delivered by B7 proteins (CD80/CD86) on activated APCs to the CD28 receptor on T cells (21). The simultaneous delivery of both signals triggers T-cell proliferation, survival, and activation. Conversely, TCR engagement in the absence of a co-stimulatory signal (e.g., from non-activated APCs) results in T-cell anergy or apoptosis (Figure 1) (22).

CTLA-4 is a regulatory receptor naturally expressed by T cells in the days following their activation. It has a much higher affinity for B7 molecules than CD28 and dislodges it in a competitive manner (23). CTLA-4 interrupts the second signal and activates regulatory signals that serve as a homeostatic negative feedback mechanism to downregulate B and T-cell activation (19, 24). The goal of abatacept is to sequester B7 molecules prior to T-cell activation and prevent the delivery of the second signal.

**Figure 2:**
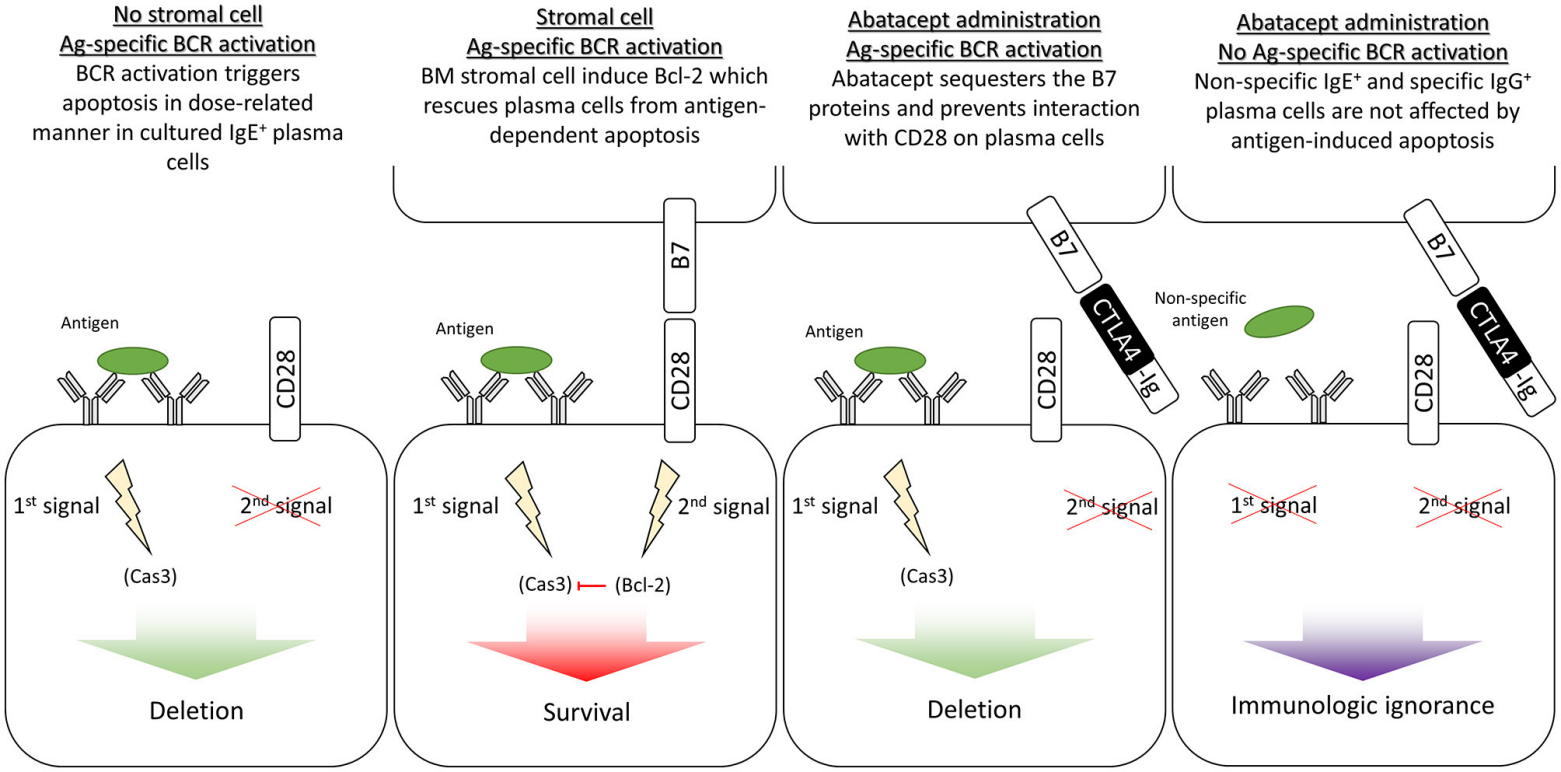
Second signal blockade in IgE^+^ long-lived plasma cells. Ag, antigen; APC, antigen-presenting cell; BM, bone marrow; Cas3, caspase-3; DC, dendritic cell; LLPC, long-lived plasma cell; MHC, major histocompatibility complex; TCR, T-cell receptor.

The concept of first and second signals also applies to some extent to IgE-producing plasma cells. Contrarily to IgG-producing plasma cells, IgE-producing plasma cells retain a functional BCR after differentiation (25), and it was recently shown that binding their antigen induces apoptosis in a dose-dependent manner (26). Plasma cells are rescued from this antigen-dependent apoptosis by CD28-induced transgene, Bcl-2 (27). Plasma cells (contrarily to B cells) express CD28, which interacts with B7 molecules on bone marrow stromal cells to prevent apoptosis (27). If abatacept was co-administered along with the antigen, B7 sequestering would be expected to prevent stromal cells from rescuing antigen-specific IgE-producing plasma cells and lead to their selective deletion (Figure 2).

### 1.4. Effect of CTLA-4 and abatacept on the allergic response

Genetic association studies have hinted at a link between CTLA-4 and allergic disease for some time. In 2004, four single nucleotide polymorphisms of CTLA-4 were found to be associated with IgE production and allergic phenotype in a cohort of 364 asthmatic families from European countries (28). The results were later confirmed in a meta-analysis regrouping 6,378 cases of asthma and 8,674 controls (29).

In 2013, a randomized controlled trial in adults with mild asthma failed to show the superiority of a 3-month treatment with abatacept over placebo at decreasing eosinophilic inflammation on subsequent allergen-specific bronchial challenges. This led the authors to conclude a lack of efficacy of second signal blockade in allergic inflammation (30). However, the study had a fundamental limitation, in that the subjects were not exposed to their allergen during the treatment phase. The results simply confirm that in the absence of the first signal, it is futile to block the second signal (immunological ignorance) (31). In auto-immune disease and transplantation, the antigen is expressed constitutively by the organism or the graft. On the other hand, allergic patients usually do their best to avoid contact with their allergen. The only way by which abatacept would be expected to exert an immunomodulatory effect in allergic disease would be if it was co-administered with the allergen.

Van Wijk et al. reported, in 2007, that abatacept completely prevented primary humoral (IgE and IgG) and cellular [interleukins (IL)-4 and 5] sensitization to peanut in mice when added to cholera toxin-based oral sensitization protocol (32). Allergic sensitization could also be prevented by pre-treatment with peanut alone prior to the oral sensitization protocol, assumedly due to the development of a primary regulatory response. Blockade of CTLA-4 during this peanut pre-treatment completely abrogated this protective effect, while blockade of IL-10 or TGF-β did not (32). Similarly, Krempski et al. recently reported that CTLA-4 blockade prevents the development of oral tolerance to peanut butter in mice (33).

Several groups reported improvements in airway eosinophil infiltration, IgE production, type-2 cytokine release, and allergen-specific airway hyper-reactivity in mice following treatment with abatacept (34–37). All animals in these studies were exposed to the allergen at some point while receiving the drug. In 2013, Maazi et al. explored the effect of combining abatacept with subcutaneous immunotherapy (SCIT) in mice sensitized to ovalbumin (38). Mice in the SCIT + abatacept treatment group had a significant improvement in allergen-induced airway hyper-reactivity, airway eosinophilia, and serum ovalbumin sIgE levels compared with mice on SCIT alone (38). Importantly, these outcomes were measured after 20 days of treatment discontinuation and thus represented sustained changes.

### 1.5. General hypothesis

We hypothesize that in teenagers and adults with persistent severe peanut allergy, administering peanut antigen through OIT while simultaneously blocking the second signal with abatacept will prevent the reactivation of the secondary allergic immune response and selectively suppress peanut-specific memory T and IgE-producing plasma cells, and thereby improve the rates of clinical remission.

The ATARI trial is a phase 2a proof-of-concept trial comparing the efficacy of abatacept vs. placebo at suppressing the sIgE surge in the 1st month of peanut OIT, treated as a proxy measure of immune memory reactivation.

## 2. Methods and analysis

### 2.1. Design

**Figure 3:**
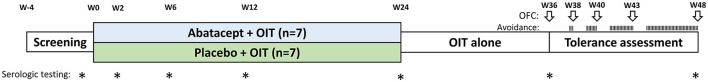
ATARI study design. OIT, oral immunotherapy; OFC, oral food challenge. *Corresponds to the serologic tests.

This study is a phase 2a, double-blinded, and randomized proof-of-concept trial comparing abatacept with placebo as an adjuvant to OIT in peanut-allergic adolescents and adults. The study will be conducted in Sainte-Justine Hospital (Montreal, Quebec, Canada). A total of 14 patients will be recruited and randomized 1:1 to receive abatacept or placebo (normal saline solution), in association with a peanut OIT for 24 weeks, after which OIT will be continued alone (Figure 3).

Starting at week 36, participants will undergo a series of up to five oral food challenges (OFCs), interlaced with incremental periods of avoidance over 12 weeks to characterize the extent of sustained unresponsiveness (SU) to peanut (Figure 3).

### 2.2. Study outcomes

The primary outcome of the trial is the relative change from baseline in peanut-specific/total IgE ratio at the end of the treatment phase (week 24). The use of the specific/total IgE ratio aims to control for non-specific fluctuations in IgE production.

Secondary outcomes include the following:

1. The relative change in peanut-specific IgG4/IgE (sIgG4/sIgE) ratio from baseline to week 24.
2. The absolute change in peanut sIgG4 from baseline to week 24.
3. The maximum period of avoidance after which an OFC with 300 mg peanut protein is still tolerated.
4. The mean cumulative function of food dosing allergic reactions.
5. The highest tolerated dose on week 36 OFC.
6. The time from the onset of OIT to the maintenance dose of 300 mg.
7. The overall rate of adverse events (AEs).

Exploratory outcomes include the following:

a. Change in peanut atopy patch test from baseline to weeks 12, 24, and 48.
b. Change in peanut skin test from baseline to weeks 12, 24, and 48.
c. Relative change in peanut-specific/total IgE from baseline to weeks 2, 6, 12, 36, and 48.
d. Relative change in peanut sIgG4/sIgE ratio from baseline to weeks 2, 6, 12, 36, and 48.
e. Absolute change from baseline in peanut sIgG4 to weeks 2, 6, 12, 36, and 48.
f. The highest tolerated dose on week 38 OFC [72 h SU].
g. The highest tolerated dose on week 40 OFC [1-week SU].
h. The highest tolerated dose on week 43 OFC [2-week SU].
i. The highest tolerated dose on week 48 OFC [4-week SU].

### 2.3. Selection of participants

The study aims to enroll 14 participants aged 14–50 years with serum peanut sIgE >50 kU/L. The inclusion and exclusion criteria are presented in Tables 1, 2, respectively. For female participants of child-bearing potential, a urine pregnancy test will be conducted on randomization day. Since asthma is an important concern when initiating OIT, a focus on uncontrolled asthma should be made. Criteria for uncontrolled asthma are as follows:

- History of two or more systemic corticosteroid courses within 6 months of screening or 1 course of systemic corticosteroids within 3 months of screening to treat asthma/wheezing;
- One hospitalization or emergency department visit for asthma/wheezing within 6 months of screening;
- Forced expiratory volume in 1 s (FEV1) <80% of predicted or FEV1/forced vital capacity (FVC) <75%, with or without controller medications;
- Inhaled corticosteroid dosing of >500 mcg daily fluticasone (or equivalent).

**Table 1:** Inclusion criteria.

1. Male or female participants 14–50 years old at screening visit.
2. History of IgE-mediated allergy to peanut protein.
3. Peanut-specific IgE level >50 kU/L.
4. Total IgE level <5,000 kU/L.
5. Willing to comply with all study requirements during participation in the study.
6. Signed informed consent and assent.

**Table 2:** Exclusion criteria.

1. Previous adverse reactions to abatacept.
2. Known hypersensitivity to abatacept or any of its components.
3. Patients at risk of sepsis, such as immunocompromised or HIV positive.
4. Patient undergoing treatment with any other biologic agent.
5. Partly controlled or uncontrolled asthma, as defined by GINA 2020.
6. Unstable angina, significant arrhythmia, uncontrolled hypertension, chronic sinusitis, or other chronic or immunological diseases that, in the judgment of the investigator, might interfere with the evaluation, administration of the test drug, or pose additional risk to the subject (e.g., gastrointestinal or gastroesophageal disease, chronic infections, scleroderma, hepatic and gallbladder disease, and chronic non-allergic pulmonary disease).
7. Current users of oral, intramuscular, or intravenous corticosteroids, tricyclic antidepressants, or beta-blocker.
8. Concurrent/prior use of immunomodulatory therapy (within 6 months), including allergen-specific immunotherapy.
9. A diagnosis of eosinophilic esophagitis, eosinophilic colitis, or eosinophilic gastritis.
10. Pregnant or breastfeeding women.
11. Chronic or latent infections with Hepatitis B, Hepatitis C, or tuberculosis.
12. Active infection.
13. Chronic Obstructive Pulmonary Disease (COPD).
14. Expected need for live vaccination during the course of the study.
15. Known malignancy.

Several study requirements will also be verified before inclusion as follows:

- Participants should be willing to be trained on the proper use of an epinephrine autoinjector and willing to possess an epinephrine autoinjector for the duration of the study or similar.
- Participants should be able to discontinue their usual anti-allergic medication prior to skin prick tests and/or OFC, as described in Section 3.7.
- Female participants with childbearing potential must agree to remain abstinent (refrain from heterosexual intercourse) or use acceptable contraceptive methods (i.e., barrier methods or oral, injected, or implanted hormonal methods of contraception or other forms of hormonal contraception that have comparable efficacy) during the trial.

### 2.4. Study drug

The first three doses of abatacept vs. placebo will be separated by an interval of 2 weeks, while the remaining doses will be given monthly, as recommended for autoimmune disorders. Since participants will be aged 14 years and older, adult dosages will be used, based on the participant's body weight: 500 mg if body weight < 60 kg, 750 mg if body weight ranges between 60 and 100 kg, and 1,000 mg if body weight > 100 kg.

The randomization list will be generated by the study pharmacist using simple randomization. The medication or placebo will be transferred into a generic IV bag to maintain blinding and identified with the participant's ID and a blinded investigational product label. It will be administered intravenously through a 0.2–1.2 μm low protein-binding filter over 30 min by the blinded study nurse, as recommended by the abatacept product monograph. Since the study drug contains maltose and the placebo does not, to ensure the blinding in participants with diabetes, glycemia will not be monitored for 4 h following the perfusion.

### 2.5. Peanut oral immunotherapy

#### 2.5.1. Initial food escalation

Peanut OIT will be initiated 24 to 72 h after the first injection of either abatacept or placebo. The initial food escalation (IFE) will consist of an OFC of up to 300 mg of peanut protein. IFE doses (presented in Table 3) will be administered every 30 min up to the target maintenance dose of 300 mg or when stopping criteria are met.

**Table 3:** Initial food escalation (IFE) up-dosing schedule.

**Dose administered (mg protein)**	**Cumulative dose (mg protein)**	**Observation time (minutes)**
1	1	30
3	4	30
10	14	30
30	44	30
100	144	30
300	444	120

Table 4 summarizes the OFC stopping rules, adapted from PRACTALL (39, 40), and details are presented in Supplementary Table 1. As a singularity in this protocol, subjective gastrointestinal symptoms, that are frequent during OFC, can be considered as possible conditions to stop OFC if they induce changes in behaviors. In case of subjective symptoms or objective signs that do not meet the stopping criteria, the investigator can delay the next dose (with a maximum of 45 min between doses) or repeat the same dose. A maximum of one dose can be repeated. In case of symptoms that do not appear in the PRACTALL guidance table, the investigator or medical staff will use their judgment to decide whether the OFC can be pursued or should be stopped. The investigator or medical staff will also decide how to effectively treat the reactions.

**Table 4:** Stopping rules for oral food challenges.

**Category**	**Symptoms**	**Grade**			
I. Skin	A. Erythematous rash: % area involved
	B. Pruritus	0	1	2	3
	C. Urticaria	0	1	2	3
	D. Angioedema	0	1	2	3
II. Upper respiratory	A. Sneezing/Itching	0	1	2	3
	B. Nasal congestion	0	1	2	3
	C. Rhinorrhea	0	1	2	3
	D. Laryngeal	0	1	2	3
III. Lower respiratory	A. Wheezing	0	1	2	3
IV. Gastrointestinal	A. Subjective complaints	0	1	2	3
	B. Objective Complaints	0	1	2	3
V. Cardiovascular or neurologic		0	1	2	3

The challenge will be stopped when any symptom reaches the red level or when two symptoms from different categories reach a yellow level. Adapted from PRACTALL (39).

#### 2.5.2. Symptom-driven up-dosing

Subjects will begin daily home dosing with the highest tolerated dose from the IFE. The highest tolerated dose is defined as the highest dose that the investigator judges safe for the patient to take at home the following day. This is generally the dose immediately before the eliciting dose, but it can be a lower dose, depending on the severity of the reaction, as per the investigator's judgment.

Participants will return to the clinic every 2 weeks for a supervised escalation of their daily peanut dose. To be eligible for up-dosing, the participant must have taken his/her full dose at least 10 times in the last 14 days. If the participant does not meet this criterion, whether it was due to cofactor prevention, logistical issues (travels, family/work event), or forgetfulness, the up-dosing visit will be postponed until the criteria are met. Participants will be informed that compliance rates lower than 80% will lead to their exclusion from the OIT program for safety reasons.

Up-dosing will also be postponed in participants in whom it is contraindicated due to active illness or allergies.

Before up-dosing, the participant's dosing diary will be reviewed by the investigator to grade dose-related reactions using the revised CoFAR Grading Scale for Systemic Allergic Reactions (version 3.0) (41). By default, the first up-dosing will plan for a +100% increase in the currently taken dose. The planned percentage increase will then be adjusted based on dose tolerance at home, following the rules presented in Table 5. If the new dose is tolerated, the following visit will plan for the same percentage increase, to be adjusted the day of, according to the rules (Table 5).

**Table 5:** Dose escalation management based on clinical tolerance.

**Home dosing symptoms during the last 2 weeks**	**Dose escalation management**
Persistent moderate (CoFAR Grade 2) or any severe (CoFAR Grade 3) or systemic reactions	No increase/next percent increase decreased by half on the following visit
Persistent mild (CoFAR Grade 1) or transient moderate (CoFAR Grade 2) local reactions	Decreased planned percent increase by half
Transient local symptoms (CoFAR Grade 1)	Proceed as planned
No or minimal symptoms^†^	Double planned percent increase

† Minimal symptoms refer to symptoms that the patient notice but does not describe as bothersome/not clinically significant, such as tingling of the tongue.

Participants who react to their dose escalation will not increase their daily dose and remain on the previously tolerated dose for another 2 weeks. The following up-dosing visit will plan for an escalation at half of the percentage increase of the failed escalation, to be adjusted based on the symptom diary. If escalation fails again, the following planned percentage increase will again be decreased by half at the subsequent visit and each following visit until the escalation is tolerated.

When the participant presents no symptoms at all to their daily dose, the up-dosing rules dictate doubling the percentage increase. However, if this would lead to an increase by a percentage up-dosing that had failed in the past, the participant is required to have two up-dosing visits with absolutely no symptoms in their diaries before proceeding to that new percentage increase, instead of just one.

Up-dosing visits will take place up to a maintenance dose of 300 mg, at which point the participant will remain on that daily dosage until the end of the study.

#### 2.5.3. Dose intake, cofactors, and dose adjustments at home

The participants will be provided with pre-weighted peanut flour doses and instructed to ingest their dose every day at around the same time. Participants will have 24 h access to research staff for support with home dosing and to guide interventions in case of adverse events. They will be trained in the recognition of anaphylaxis and cofactors that increase the risk of reactions when they are taking their dose (e.g., alcohol, exercise, and infections). They will be prescribed an epinephrine auto-injector and will be trained on its use.

Medication can be prescribed to prevent OIT-induced allergic symptoms with daily dosing, as per the investigator's discretion. This can include H1 and/or H2 anti-histamine, leukotriene receptor antagonists, proton-pump inhibitors, prostaglandin E1 analogs, mast cell stabilizers, and/or swallowed corticosteroids. These medications will be documented in the patient diary and concomitant medication log.

In the event of an identifiable cofactor (e.g., active viral infection), the OIT dose would be temporarily decreased by half. The participant would resume full dosing once the cofactor has been resolved. In the event of any systemic reaction or a moderate-to-severe local reaction at home, the OIT dose would be decreased by half until the following planned up-dosing visit.

### 2.6. Oral tolerance testing phase

Oral tolerance will be assessed starting at week 36 to allow for a 4-month washout period after the last abatacept or placebo perfusion (Figure 3). Participants will undergo an OFC of up to 9,000 mg of peanut protein, corresponding to one serving of peanuts (1/4 cup), aimed at testing the extent of desensitization while on therapy. OFC-stopping rules are presented in Table 4 and Supplementary Table 1.

#### 2.6.1. 72 h SU testing (w38)

Participants who tolerate at least 300 mg of peanut protein on week 36 OFC will proceed to SU testing. They will be asked to continue daily dosing with 300 mg of peanut protein for 11 days, after which they will avoid peanuts for 72 h and then return to the clinic to repeat an OFC up to 9,000 mg of peanut protein.

#### 2.6.2. One-week SU testing (w40)

Participants who tolerate 300 mg of peanut protein on 72 h SU testing will resume daily dosing for 1 week and then avoid peanuts for a whole week, before returning to the clinic to repeat the OFC up to 9,000 mg peanut protein.

#### 2.6.3. Two-week SU testing (w43)

Participants who tolerate 300 mg of peanut protein on the 1-week SU testing will resume daily dosing for 1 week and then avoid peanuts for 2 weeks, before returning to the clinic to repeat the OFC up to 9,000 mg peanut protein (2w SU).

#### 2.6.4. Four-week SU testing (w48)

Participants who tolerate 300 mg of peanut protein on the 2-week SU testing will resume daily dosing for 1 week and then avoid peanuts for 4 weeks, before returning to the clinic for a final OFC up to 9,000 mg peanut protein on week 48.

At any time in the SU testing phase of the study, if a participant presents symptoms to the dose of 300 mg, they will resume peanut OIT and continue daily dosing with no further SU testing until the end of the study at week 48.

### 2.7. Prohibited prior and concomitant medication

At any time during the study, participants should not take any of the following treatments:

- Another investigational drug or approved therapy for investigational use;
- Any biological immunomodulatory therapy or anti-tumor necrosis factor drug;
- Any concomitant immunotherapy administered to any food;
- Any aeroallergen or venom immunotherapy initiated or in the up-dosing phase during study participation;
- Systemic steroids (intravenous, intramuscular, or oral dosing) for more than 7 days;
- Beta-blocking agents.

Before OFCs, subjects will be asked to stop the use of antihistamines (72 h for short-acting and 5–7 days for long-acting) and theophylline (12 h).

Before skin prick testing, subjects will be asked to stop the use of antihistamines (72 h for short-acting and 7 days for long-acting).

### 2.8. Safety assessments

All adverse events (AEs) occurring during the study will be documented in the e-CRF. Concomitant illnesses, which existed before entry into the study, will not be considered AEs unless they worsen during the treatment period. All AEs, regardless of the source of identification (for example, physical examination, laboratory assessment, and reported by subject), will be documented, as well as their severity, seriousness, and causality. AEs attributed to food dosing during OIT will be treated as AEs of special interest (AESI). IgE-mediated reactions related to OIT dosing will be recorded separately from the AE log following the CoFAR grading system.

### 2.9. Statistical considerations

Based on observations from preclinical studies in mice, abatacept is expected to suppress the allergen-specific IgE surge induced by OIT. The relative change in specific/total IgE at 24 weeks will be compared between the two groups using Student's *t*-test. A 50% suppression was deemed clinically significant.

Based on the PALISADE trial (6), we expect the placebo group (OIT alone) to increase their specific IgE levels by 100% with a standard deviation of <30%. Based on this assumption, a sample size of 12 participants with a 1:1 randomization ratio would allow the detection of a 50% difference in the increase in sIgE, with an alpha risk of 0.05 and a power of 80%. A total sample size of 14 patients would allow to account for 15% of lost follow-up.

## 3. Discussion

The hypothesis tested in the ATARI study is that abatacept combined with peanut OIT will lead to peanut-specific T and plasma cell suppression and thus increase the rates of clinical remission compared with peanut OIT alone. The hypothesis has strong biological plausibility and is supported by animal models, but it has never been tested in humans.

### 3.1. On the choice of proxy outcome

ATARI is intended as a proof-of-concept study and will likely not be powered to detect differences in clinical remission. Its primary aim is, therefore, to assess differences in a proxy outcome that would be associated with the development of clinical remission.

Based on the choice of proxy outcome, we observed from preclinical studies that abatacept suppresses the initial surge in sIgE with allergen exposure (32). This is assumed to be the consequence of the suppression of peanut sIgE-producing plasma cells and T-cell reactivation and is shown to correlate with the likelihood of clinical remission (9, 17, 18). If this key finding cannot be replicated in humans, it appears reasonable to assume that the likelihood of replicating the clinical effects would also be low.

One risk of using the surge in sIgE as a primary outcome in such a small sample is that we are highly vulnerable to random non-specific variations in total IgE productions, which could have a confounding effect and decrease power. In our own experience of longitudinal testing during peanut OIT or epicutaneous immunotherapy, spontaneous variations in sIgE, even large, are non-clinically significant when they parallel variations in total IgE, hence the choice to measure changes in the sIgE over total IgE ratio as the primary outcome.

Changes in the sIgG4/sIgE ratio are another paraclinical outcome that is often measured during OIT. Since IgG4 production follows the same non-specific expansion/contraction variations as IgE, it is generally a more reliable measure of longitudinal changes in OIT than sIgE alone. However, it is unclear how abatacept will affect sIgG4 production. Based on preclinical data, it is possible that it may suppress the rise in sIgG4, which would complicate interpretation. The sIgG4/sIgE ratio will, therefore, be treated as a secondary outcome.

### 3.2. On sustained tolerance testing

In addition to providing the “go/no-go” decision for a subsequent larger trial testing clinical remission, ATARI provides a unique opportunity to pilot the design for such a trial. In previous OIT studies, clinical remission had been tested using fixed durations of food avoidance, which could last up to 6 months (9, 42). While very long periods of avoidance are interesting from a conceptual point-of-view, our patient partners felt that avoidance beyond 4 weeks is less relevant to them and that the higher risk of losing hard-earned desensitization during prolonged avoidance is an important barrier. In fact, even the 4-week avoidance period is found to have low acceptability, despite its relevance.

This has led to the current design of the sustained tolerance testing phase, where OFCs are performed after a series of incremental periods of avoidance, each separated by periods of daily dosing. While the initial intent is to increase patient acceptability, an additional upside is that it provides tolerance thresholds after a wide number of avoidance periods, which is highly relevant data for patients and clinicians. The downside of the approach is the need for multiple OFC visits. In the end, the ATARI trial will provide precious information on the feasibility and acceptability of this design.

### 3.3. On treatment acceptability

The trial will also inform as to the acceptability of the medication itself. Most biologics used in allergy have had very good safety profiles, mostly without any immunosuppression. While abatacept is not considered immunosuppressive *per se*, it can still impede physiological immune responses and increase the risk of infection while on therapy, especially if combined with methotrexate and in patients with chronic obstructive pulmonary disease (COPD) (43). Immunosuppressive medication, COPD, and active infection are exclusion criteria in ATARI. The screening will include mandatory testing for hepatitis B and C, HIV, and tuberculosis.

### 3.4. On oral food challenges

In ATARI, we chose not to confirm peanut allergy by OFC prior to randomization. The rationale was to avoid that the first contact with peanut allergen would occur without a second signal blockade, which could theoretically prime the allergic response and act as a confounder. Whether this would be the case or not is unknown, but given this is a proof-of-concept trial, we chose to err on the side of safety (3, 5, 44). This comes with a risk of randomizing patients that are found to be tolerant to 300 mg of peanut protein 2 days later on the initial food escalation OFC. Since all patients will have sIgE of at least 50 kU/L, we would still be able to assess changes in the humoral response to OIT. If patients have a baseline threshold above 300 mg, the consequence would be a loss in power for the sustained tolerance outcomes. The study will help inform an eventual phase 3 trial regarding this risk.

We also decided not to exclude patients who underwent a recent known or suspected IgE-mediated reaction to peanuts to avoid any selection bias. Such a “real-life” condition is different from intentional exposure to peanuts with OFCs and appeared acceptable. We will then document the history of previous exposure to peanuts, and we should be able to control for this to some degree in the analyses.

All OFCs in ATARI are open-label. Because the OFCs need to take place at very precise moments in the schedule (24 h after randomization and immediately at the end of periods of avoidance), double-blind placebo-controlled food challenges occurring on 2 different days, up to a week apart, would not have been compatible with the study design.

From a methodological point of view, since patients will be effectively blinded to their treatment arm, both groups should be equally affected by the risk of the subjectivity of open-label OFCs. Therefore, the only tangible impact on study integrity would be a loss in power for OFC-dependent outcomes. This can be compensated by adjusting the sample size in an eventual phase 3 trial.

### 3.5. On the choice of IV abatacept

The study uses the currently approved intravenous dosages of abatacept for autoimmune disorders. While subcutaneous injections may be more practical in real-life, product viscosity and the possibility of local reactions make them more difficult to blind in a research setting. Another argument for intravenous administration was the rapid distribution of the medication, which increases the likelihood of having reached therapeutic levels in lymphoid tissue by the time OIT is initiated 24–72 h later.

### 3.6. On the up-dosing protocol

The advantage of the symptom-driven OIT protocol compared with fixed schedules is that it allows faster progression when tolerated. This maximizes the time spent at full maintenance dose while the investigational product is at therapeutic levels. This may or may not be clinically relevant. The minimal amount of peanut protein needed to induce remission with or without abatacept is currently unknown, but experience with epicutaneous and sublingual immunotherapy suggests that it may actually be quite low (40, 45).

## 4. Conclusion

The ATARI study will provide the first human data on the use of abatacept as an adjuvant to allergen immunotherapy to promote sustained tolerance. The study uses an original design taking into account various biological, clinical, and patient considerations specific to abatacept or tolerance testing, and will aid in the conduction of future large-scale trials.

## Ethics statement

The studies involving human participants were reviewed and approved by Comité d'éthique du CHU Sainte-Justine. Written informed consent to participate in this study was provided by the participants' legal guardian/next of kin.

## Author contributions

CB and PA contributed to protocol and manuscript writing. CB, FG-L, ÉR, KS, FG, LP, AD, and PB contributed to investigations. PB elaborated the design of the study and promoted the study. All authors contributed to the article and approved the submitted version.
