# Supplementary material for: Abatacept to induce remission of peanut allergy during oral immunotherapy (ATARI): protocol for a phase 2a randomized controlled trial
Source: Front Med (Lausanne). 2023 Jun 28;10:1198173. doi: 10.3389/fmed.2023.1198173 (PMC10336224; doi:10.3389/fmed.2023.1198173)
Supplement: Supplementary file 1 [file Table_1.docx]

**Supplementary Table 1. Detailed stopping rules for oral food challenges.** Challenge will be stopped when any symptom reaches the red level or when two symptoms from different categories reach a yellow level. Adapted from PRACTALL (39).

| **Category** | **Symptoms** | **Grade** |
| --- | --- | --- |
| **I. Skin** | A. Erythematous rash: % area involved | 0= Absent  1= Mild – A few areas of faint erythema  2= Moderate – Areas of erythema (> 20% and <50%), macular and raised rash  3= Severe – generalized erythema (> 50%), extensive raised lesions (> 25%) |
|  | B. Pruritus | 0= Absent  1= Mild, occasional scratching  2= Moderate scratching continuously for > 2 minutes at a time  3= Severe – hard continuous scratching – excoriations |
|  | C. Urticaria | 0= Absent  1= Mild - ≤ 3 hives  2= Moderate - < 10 hives but > 3  3= Severe – generalized involvement |
|  | D. Angioedema | 0= Absent  1= Mild –mild lip edema  2= Moderate – significant face or lip edema  3= Severe – generalized involvement |
| **II. Upper Respiratory** | A. Sneezing/Itching | 0= Absent  1= Mild rare – rare bursts  2= Moderate – bursts < 10, intermittent rubbing of nose, external ear canals and/or eyes  3= Severe – continuous rubbing of nose and/or eyes, periocular swelling and/or long bursts of sneezing |
|  | B. Nasal congestion | 0= Absent  1= Mild – some hindrance to breathing  2= Moderate – nostrils feel blocked, breathing through mouth most of the time  3= Severe – nostrils occluded |
|  | C. Rhinorrhea | 0= Absent  1= Mild – occasional sniffling  2= Moderate - frequent sniffling, requires tissues  3= Severe – nose runs freely despite sniffling and tissues |
|  | D. Laryngeal | 0= Absent  1= Mild – Grade 1  2= Moderate – Grade 2  3= Severe – Grade 3 |
| **III. Lower Respiratory** | A. Wheezing | 0= Absent  1= Mild – Expiratory wheezing to auscultation  2= Moderate – dyspnea, inspiratory and expiratory wheezing  3= Severe – dyspnea, use of accessory muscles, audible wheezing |
| **IV. Gastrointestinal** | A. Subjective complaints: abdominal pain or nausea | 0= Absent  1= Mild, no change in activity  2= Moderate, associated with decreased activity  3= Severe and/or persistent, associated with significant change in behaviour |
|  | B. Objective Complaints: emesis or diarrhea | 0= Absent  1= Mild – 1 episode of emesis or diarrhea  2= Moderate - 2-3 episodes of emesis or diarrhea or 1 of each  3= Severe - > 3 episodes of emesis or diarrhea or 2 of each |
| **V. Cardiovascular or neurologic** | A. Cardiovascular/neurologic | 0= Normal heat rate or BP for age/baseline  1= Mild - color change, subjective response (weak, dizzy), or tachycardia, mental status change, mild hypotension (weak rapid pulse and/or 10-20% drop in blood pressure from baseline)  2= Moderate – drop in blood pressure >20 % from baseline, significant change in mental status, light-headedness, feeling of pending doom  3= Severe – cardiovascular collapse, sign of impaired circulation, unconsciousness, bradycardia, cardiac arrest |
